# Supplementary material for: The SNAP-25 linker supports fusion intermediates by local lipid interactions
Source: eLife. 2019 Mar 18;8:e41720. doi: 10.7554/eLife.41720 (PMC6422494; doi:10.7554/eLife.41720)
Supplement: Transparent reporting form [file elife-41720-transrepform.docx]

***eLife’s* transparent reporting form**

We encourage authors to provide detailed information *within their submission* to facilitate the interpretation and replication of experiments. Authors can upload supporting documentation to indicate the use of appropriate reporting guidelines for health-related research (see [EQUATOR Network](http://www.equator-network.org/%20)), life science research (see the [BioSharing Information Resource](https://biosharing.org/)), or the [ARRIVE guidelines](http://www.plosbiology.org/article/info:doi/10.1371/journal.pbio.1000412) for reporting work involving animal research. Where applicable, authors should refer to any relevant reporting standards documents in this form.

If you have any questions, please consult our Journal Policies and/or contact us: [editorial@elifesciences.org](mailto:editorial@elifesciences.org).

**Sample-size estimation**

- You should state whether an appropriate sample size was computed when the study was being designed
- You should state the statistical method of sample size computation and any required assumptions
- If no explicit power analysis was used, you should describe how you decided what sample (replicate) size (number) to use

Please outline where this information can be found within the submission (e.g., sections or figure legends), or explain why this information doesn’t apply to your submission:

Information about sample size is given in the ‘Statistics’ subsection of ‘Material and Methods’. Please note that the computation of sample size estimates is not commonly performed for the types of experiments (electrophysiological recordings in cultured cells and biochemical assays) presented in our study.

**Replicates**

- You should report how often each experiment was performed
- You should include a definition of biological versus technical replication
- The data obtained should be provided and sufficient information should be provided to indicate the number of independent biological and/or technical replicates
- If you encountered any outliers, you should describe how these were handled
- Criteria for exclusion/inclusion of data should be clearly stated
- High-throughput sequence data should be uploaded before submission, with a private link for reviewers provided (these are available from both GEO and ArrayExpress)

Please outline where this information can be found within the submission (e.g., sections or figure legends), or explain why this information doesn’t apply to your submission:

The number of replicates is indicated in corresponding figure panels.

Explanations on data handling, biological replicates, and exclusion criteria can be found in ‘Materials and Methods’, especially in the subsection ‘Statics’. For information on data collection in electrophysiological experiments, please also refer to the subsection ‘Cell culture and electrophysiological recordings’.

**Statistical reporting**

- Statistical analysis methods should be described and justified
- Raw data should be presented in figures whenever informative to do so (typically when N per group is less than 10)
- For each experiment, you should identify the statistical tests used, exact values of N, definitions of center, methods of multiple test correction, and dispersion and precision measures (e.g., mean, median, SD, SEM, confidence intervals; and, for the major substantive results, a measure of effect size (e.g., Pearson's r, Cohen's d)
- Report exact p-values wherever possible alongside the summary statistics and 95% confidence intervals. These should be reported for all key questions and not only when the p-value is less than 0.05.

Please outline where this information can be found within the submission (e.g., sections or figure legends), or explain why this information doesn’t apply to your submission:

Sample size, statistical significance, and employed statistical analysis method are indicated within figures and corresponding legends. Information on the statistical analysis methods is also given in the ‘statistics’ subsection of ‘Materials and Methods’. Raw data are depicted wherever possible (exemplary gel pictures and blots in Fig. 2A,B, Fig.2 – Figure Supplement 1, and Fig. 3 –Figure Supplement 1; pictures of Western Blots in Fig.1 – Figure Supplement 1; microphotographs in Fig. 4A, Fig.1 – Figure Supplement 2/3, and Fig.3 –Figure Supplement 1A; single amperometric spikes in Fig. 6A). For transparency, statistical summaries are provided as source data for all figures.

(For large datasets, or papers with a very large number of statistical tests, you may upload a single table file with tests, Ns, etc., with reference to sections in the manuscript.)

**Group allocation**

- Indicate how samples were allocated into experimental groups (in the case of clinical studies, please specify allocation to treatment method); if randomization was used, please also state if restricted randomization was applied
- Indicate if masking was used during group allocation, data collection and/or data analysis

Please outline where this information can be found within the submission (e.g., sections or figure legends), or explain why this information doesn’t apply to your submission:

Not applicable. Our study does not present data from clinical trials or other experiments that would benefit from systematic sample randomization.

**Additional data files (“source data”)**

- We encourage you to upload relevant additional data files, such as numerical data that are represented as a graph in a figure, or as a summary table
- Where provided, these should be in the most useful format, and they can be uploaded as “Source data” files linked to a main figure or table
- Include model definition files including the full list of parameters used
- Include code used for data analysis (e.g., R, MatLab)
- Avoid stating that data files are “available upon request”

Please indicate the figures or tables for which source data files have been provided:

Statistical summary tables have been uploaded as source data for all figures.
